# Biomimetic Nanozymes Suppressed Ferroptosis to Ameliorate Doxorubicin-Induced Cardiotoxicity via Synergetic Effect of Antioxidant Stress and GPX4 Restoration

**DOI:** 10.3390/nu15051090

**Published:** 2023-02-22

**Authors:** Yunpeng Zhang, Shuang Liu, Jing Peng, Shifeng Cheng, Qingling Zhang, Nan Zhang, Zandong Zhou, Yue Zhang, Yang Zhao, Tong Liu

**Affiliations:** 1 Department of Cardiology, The Second Hospital of Tianjin Medical University, Tianjin 300211, China; 2 Tianjin Key Laboratory of Ionic-Molecular Function of Cardiovascular Disease, Tianjin Institute of Cardiology, The Second Hospital of Tianjin Medical University, Tianjin 300211, China; 3 Department of Radiology, The Second Hospital of Tianjin Medical University, Tianjin 300211, China

**Keywords:** doxorubicin-induced cardiomyopathy, ferroptosis, mitochondria, nanozyme, oxidative stress, biomineralization

## Abstract

Mitochondria-dependent ferroptosis plays an important role in the pathogenesis of doxorubicin (DOX)-induced cardiotoxicity (DIC), which remains a clinical challenge due to the lack of effective interventions. Cerium oxide (CeO_2_), a representative nanozyme, has attracted much attention because of its antioxidant properties. This study evaluated CeO_2_-based nanozymes for the prevention and treatment of DIC in vitro and in vivo by adding nanoparticles (NPs), which were synthesized by biomineralization, to the culture or giving them to the mice, and the ferroptosis-specific inhibitor ferrostatin-1 (Fer-1) was used as control. The prepared NPs exhibited an excellent antioxidant response and glutathione peroxidase 4 (GPX4)-depended bioregulation, with the additional merits of bio-clearance and long retention in the heart. The experiments showed that NP treatment could significantly reverse myocardial structural and electrical remodeling, and reduce myocardial necrosis. These cardioprotective therapeutic effects were associated with their ability to alleviate oxidative stress, mitochondrial lipid peroxidation, and mitochondrial membrane potential damage, with a superior efficiency to the Fer-1. The study also found that the NPs significantly restored the expression of GPX4 and mitochondrial-associated proteins, thereby restoring mitochondria-dependent ferroptosis. Therefore, the study provides some insights into the role of ferroptosis in DIC. It also shows that CeO_2_-based nanozymes could be a promising prevention and treatment candidate as a novel cardiomyocyte ferroptosis protector to mitigate DIC and improve prognosis and quality of life in cancer patients.

## 1. Introduction

A previous study has shown that 8.8% of cancer survivors die from cardiovascular diseases [1]. Multiple clinical trials and large retrospective cohort analyses have reported that more than half of cancer survivors who are treated with anthracyclines will develop a severe cardiovascular disease [2,3,4,5]. Doxorubicin (DOX), a representative of anthracyclines, is the first-line standard treatment for numerous cancers that inhibit ribonucleic acid (RNA) synthesis and deoxyribonucleic acid (DNA) replication [6,7]. However, pharmacokinetic studies have found that DOX may be cardiotoxic, leading to irreversible degenerative cardiomyopathy, congestive heart failure, and various arrhythmias, known as doxorubicin-induced cardiotoxicity (DIC). This limits the clinical applications of DOX [4,8,9,10,11,12,13,14,15]. Unfortunately, no effective alternative interventions are available for the prevention and treatment of DIC at present, although its mechanism has been extensively investigated [16,17,18,19,20,21,22,23,24].

In 2019, Fang et al. reported for the first time that ferroptosis, which is characterized by the lethal accumulation of iron-dependent lipid reactive oxygen species (ROS), is an important mechanism that leads to the development of DIC instead of apoptosis, necrosis, or pyroptosis [25]. Targeted intervention of ferroptosis with the specific inhibitors, such as ferrostatin-1 (Fer-1), could effectively prevent DIC. Tadokoro et al. reported that glutathione peroxidase 4 (GPX4) is the key regulator of ferroptosis, by constructing GPX4 overexpressing and knockout mouse models [26]. The administration of GPX4 is reported to significantly inhibit the toxicity of lipid peroxidation, while maintaining the homeostasis of the membrane lipid bilayer [27,28,29]. Based on the above, we thus hypothesize that GPX4 and its activators should have the potential to protect cancer patients against DIC by suppressing ferroptosis.

In recent years, catalytic nanozymes, as a new generation of artificial enzymes, have received much attention due to their strong antioxidant properties, easy synthesis, controllable size, and tunable function [30]. Among all such nanozymes, cerium oxide (CeO_2_), was one of the first nanoparticles (NPs) proposed for use as a therapeutic agent, because its surface presents redox cycling between the Ce^4+^ (CeO_2_) or Ce^3+^ (Ce_2_O_3_) states of cerium [31,32,33,34]. CeO_2_ NPs exerts hybrid superoxide dismutase (SOD)- and catalase (CAT)-mimetic enzyme activities, allowing the efficient protection of rat heart myoblasts H9c2 cell lines from oxidative damage [35,36,37]. Additionally, several oxygen vacancies have been identified on the surface of CeO_2_ NPs, resulting in efficient nanoenzymatic activity [38,39]. Sangomla et al. observed that CeO_2_ NPs could protect cardiomyocytes from oxidative stress in mice models of DIC [40]. Furthermore, Cai et al. reported that Ce-doped carbon dots strongly protected mice embryonic fibroblast cells from oxidative damage in erastin-mediated ferroptosis [41]. However, the potential side effects of CeO_2_ NPs, including altered alveolar function, significant lung damage, and marked cytotoxicity, limit their use in biomedical applications [42,43]. Therefore, developing biocompatible CeO_2_ NPs with an optimal surface area that can afford excellent catalytic efficiency at the minimum dose remains a scientific hurdle.

A biomineralization synthesis strategy has been associated with several advantages in the field of nanomaterial development, such as narrow size distribution, excellent biocompatibility, high stability, high yield, and low cost when compared with traditional physical and chemical methods [44,45,46,47,48]. For instance, the albumin-bound paclitaxel nanoparticle Abraxane^®^ has been approved for clinical use in treating metastatic breast cancer [49]. Additionally, Kalashnikova et al. reported on a bovine serum protein (BSA)-ceria-indocyanine green (ICG) nanoparticle for treating rheumatoid arthritis via anti-oxidative stress and anti-inflammatory action [50]. In the present study, we successfully synthesized a biomimetic CeO_2_-based nanozyme by biomineralization with proteins as the template, increasing the specific surface area and the number of oxygen vacancies. The prepared CeO_2_ exhibited both SOD- and CAT-mimetic enzyme activities. These have potential clinical use in the prevention and treatment of DIC via the rapid removal of ROS. It was also observed that the nanozyme significantly reversed cardiac structural and electrical remodeling and reduced myocardial necrosis.

**Figure 1:**
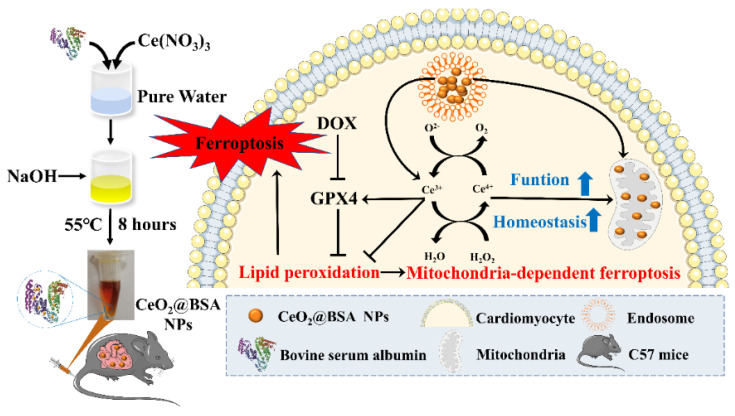
Scheme of biomimetic cerium-oxide-based nanozyme synthesis by biomineralization. After tail vein injection, the nanozymes were taken up by the cardiomyocytes and mitochondria, activating the GPX4 to reverse ferroptosis by maintaining mitochondrial function and homeostasis. BSA: bovine serum protein, NPs: nanoparticles, DOX: doxorubicin, GPX4: glutathione peroxidase 4.

Furthermore, in terms of cellular and molecular mechanisms, the experiments in this study demonstrated that nanozyme intervention not only significantly suppressed oxidative stress, mitochondrial lipid peroxidation, and mitochondrial membrane potential (MMP) damage, but also restored mitochondria-dependent ferroptosis, as well as increased GPX4 and mitochondrial-associated protein expression (Figure 1). Interestingly, the metabolism analysis of cerium by ICP-MS showed that the CeO_2_ nanozyme has longer retention in the heart compared to Ce(NO_3_)_3_ at the equivalent cerium content. Furthermore, the nanozyme could be cleared by the hepatobiliary systems. The routine blood test, liver function, kidney function, and histological evaluation of major tissues or organs showed that the nanozyme has a good biocompatibility. To the best of our knowledge, this is the first study to explore the cardioprotective effect of antioxidative cerium NPs on DIC by activating the GPX4-dependent pathways to suppress ferroptosis and maintain mitochondrial homeostasis and function, which was even superior to Fer-1. In summary, CeO_2_-based nanozymes could be clinically translated as novel antioxidants and synergetic GPX4 activators to inhibit cardiomyocyte ferroptosis, thereby mitigating DIC in cancer patients, while improving their prognosis and quality of life. The study also provides useful insights into the role of ferroptosis in DIC.

## 2. Materials and Methods

### 2.1. Drugs and Chemicals

Cerium nitrate hexahydrate [Ce(NO_3_)_3_·6H_2_O] (99.5%, Sigma, Livonia, MI, USA), bovine serum albumin [BSA (molecular weight 76–81 KD)], Dulbecco’s modified Eagle’s medium (DMEM), fetal bovine serum (FBS, Gibco, Billings, MT, USA) and penicillin-streptomycin (Gibco), 3-(4,5-Dimethylthiazol-2-yl)-2,5-Diphenyl Tetrazolium Bromide assay (MTT), DOX (D107159), and 2′,7′-dichlorofluorescin diacetate (DCFH-DA, Sigma) were supplied by Aladdin Reagent Co., Ltd. (Aladdin, Shanghai, China). Fluorescein isothiocyanate (FITC)-conjugated BSA was purchased from Solarbio Science & Technology Co., Ltd. (Solarbio, Beijing, China). Ferrostatin-1(Fer-1) was purchased from MedChemExpress (MCE, Shanghai, China). All commercial products were used without being subject to further purification.

### 2.2. Synthesis of CeO_2_@BSA NPs

Based on previous studies [27,51], this could be biomineralized within the BSA protein as the template to assist the fabrication of Ce-based NPs. Ce(NO_3_)_3_·6H_2_O (9 mg) and BSA (60 mg) were dissolved in pure water (2.4 mL) at 37 °C and stirred for 5 min. NaOH (80 mM) was slowly added to the mixture drop by drop until the reaction system reached pH ≥ 10 to induce the formation of CeO_2_ and simultaneously unfold the BSA, which resulted in the precipitation of CeO_2_ nanoparticles into the BSA. The reaction solution was then stirred at 55 °C for 8 h. The resulting CeO_2_@BSA were washed and separated by centrifugation at 12,000 rpm for 10 min to obtain a transparent supernatant. Ultrafiltration was also performed with a 30 kDa centrifugal filter unit [Amicon Ultra-15 Centrifugal Filter Unit with Ultracel-30 membrane (30 kDa), Millipore] at 5000 rpm for 10 min to remove excess Ce^3+^ in the supernatant. These purification procedures were repeated 3 times. Storage was conducted at 4 °C. The Ce content in the purified solution was determined using an X Series quadrupole inductively coupled plasma mass spectrometry instrument (ICP-MS, Thermo Elemental, Cheshire, UK). The stock solution was diluted to give the various concentrations required for the subsequent experiments. The FITC-conjugated BSA was purchased from Solarbio (SF063). A brief description of the synthesis method is as follows: 1 Labeling materials: (1) FITC isomer I, from Sigma, MW: 389.4, excitation wavelength of 495 nm, emission wavelength of 525 nm. (2) BSA, MW: ~68,000, 98% purity. (3) Labelling buffer: 0.5M pH = 9.5 carbonate buffer, eluent: 0.01 M pH =7.2 PBS. 2 Labelling method. Direct labelling method: reaction at room temperature for about 2.5–3.0 h, centrifuged through Sephadex G-50, and then washed column with 0.01M pH = 7.2 PBS. 3 Mass analysis. The solution was 0.01M pH = 7.2 PBS (containing 0.03% proclin, 50% glycerol) with a protein concentration of about 5 mg/mL and an FITC concentration of 30–45 μg/mL containing preservative. Then, the synthesis method and procedure of CeO_2_@BSA-FITC NPs was the same as CeO_2_@BSA NPs, fully protected from light. The CeO_2_@BSA-FITC NPs were stored at 4 °C away from light for cellular uptake experiments.

### 2.3. Nanoparticles Characterization

The mean hydrodynamic size distribution was characterized using a BT-90 Nanoparticle Analyzer (Better, China) at 25 °C and the zeta potentials were monitored using NanoS90 (Malvern, Worcestershire, UK) at 25 °C. A drop of CeO_2_@BSA solution was carefully applied to the carbon-coated copper grids and dried under vacuum at room temperature. The morphology of the NPs was observed with high-resolution transmission electron microscopy (HRTEM, Philips Tecnai G2 F20, Amsterdam, The Netherlands). The crystal phase of CeO_2_@BSA was further confirmed via X-ray diffraction (XRD), and X-ray photoelectron spectroscopy (XPS) was used to characterize the Ce hybrid oxidation state.

### 2.4. SOD Activity Assay and Catalase Activity Assay

The SOD-mimetic enzyme activity assay was measured according to the instructions of the manufacturer (Nanjing Jiancheng Bioengineering Institute, Nanjing, China). The catalase (CAT)-mimetic enzyme activity assay was measured according to the methods published in the previous literature [28].

### 2.5. Cellular Uptake Experiments

H9c2 cells line (Cellcook Biotech Co., Ltd., Guangzhou, China) were pre-seeded into confocal Petri dishes and incubated in DMEM cell medium supplemented with 10% FBS at 37 °C in 5% CO_2_. When the cells entered the log phase growth, the medium was replaced with 1 mL of medium containing different concentrations of the CeO_2_@BSA-FITC NPs (200 μM). After a 6 h incubation period, the cells were washed three times with PBS and observed for a fluorescent signal using an Olympus FV1000 LSCM (Tokyo, Japan) under 488 nm for excitation and 525 nm for emission.

### 2.6. Cytotoxicity Assay

Standard MTT assays were carried out to visualize the cytotoxicity of CeO_2_@BSA. H9c2 cell lines were pre-seeded into 96-well cell culture plates. When the cells entered the log phase growth, the medium was replaced with 200 µL of medium containing different concentrations of the NPs (0, 50, 100, 150, 200, 300, 400, 500, 600, 800, 1000, or 2000 µM). After 24 h of incubation, the cells were washed three times with PBS, and MTT stock solution (5 mg/mL) was added to each well and left to react for 4 h. The medium was then discarded and dimethyl sulfoxide (150 µL/well, Sigma–Aldrich, St. Louis, MO, USA) was added to each well. The assay plate was placed on a shaker (QLBE, Haimen, China) for 10 min, the optical density (OD) value for each well was recorded at 570 nm with a microplate reader (Thermo, Varioskan Flash). Cell viability was calculated using the following formula:

cell viability (%) = (mean OD of selected treatment group/mean OD of the control group) × 100.

The data are presented as the mean value with standard deviation from six independent experiments.

### 2.7. Experimental Design and Model Establishment

C57BL/6J male mice aged 8–10 weeks (average body weight ≈ 25 g) were purchased from Beijing HuaFuKang Bioscience Co., Ltd. (Beijing, China). These mice were fed under controlled conditions with a constant temperature of 25 ± 2 °C, a humidity of 60 ± 5%, and a 12 h day/night cycle, given a sufficient standard animal diet and drinking water, and the cage bedding was changed every other day. All the animals were acclimated to the environment for one week before the experiment. The mice were randomly divided into four groups: control (NS, daily intraperitoneal administered normal saline, n = 6), DOX (twice every other day i.p. administered DOX, 7.5 mg/kg, n = 6), DOX + Fer-1 (daily i.p. administered Fer-1 1 mg/kg, n = 6), and DOX + CeO_2_@BSA NPs (intravenous administration 1 mg/kg measured by cerium for total of four doses, n = 6). The animal study protocol was approved by the Experimental Animal Management Committee of the Tianjin Medical University (TMUaMEC 2019004), and the experimental operation was carried out in accordance with the guidelines of the National Institutes of Health of the United States and in line with the 3R principles.

### 2.8. Determination of MDA, GSH, NT-proBNP, and cTnI in Mice Serum

The levels of malondialdehyde (MDA) and glutathione (GSH) were measured according to the instructions of the manufacturer (Solarbio, Beijing, China). Mice ELISA kits (Wuhan Huamei Biological Engineering Co., Ltd., Wuhan, China) were used to evaluate the serum levels of the N-terminal brain natriuretic peptide (NT-proBNP) and cardiac troponin I (cTnI).

### 2.9. Echocardiography

All the mice underwent transthoracic echocardiography utilizing a Vevo 2100 system with an MS400 linear array transducer (Visual Sonics, Toronto, ON, Canada) in a blinded manner by a specialist physician. Briefly, the mice were anesthetized with isoflurane, the chest hair was removed, and the mice were placed in a supine position on a constant temperature table at 37 °C, and a small animal ultrasound system was used to record transthoracic echocardiographic images. Two-dimensional and M-mode images of the long-axis and short-axis sections of the parasternal left ventricle of the mouse heart were collected, and the LVID, LVPW, and IVS were measured. The left ventricular ejection fractions (LVEF), left ventricular fractional shortening (LVFS), and left ventricular volume (LV Vol) were calculated according to international standards. All the ultrasound parameters were based on the average of 5 consecutive cardiac cycles.

### 2.10. Surface ECG and Epicardial Electrical Mapping

After satisfactory anesthesia with 1.5% tribromoethanol (20 mg/kg), at least 10 stable continuous electrocardiography (ECG) waveforms were recorded using a multiconductive physiological recorder (TOP-2001). Then, the widths of P wave, PR interval, QRS duration, QT interval, and RR interval were measured and analyzed using LabChart 8 Reader software (LabChart Pro software, version 8; AD Instruments, Shanghai, China). After surface ECG examination, with the support of a small animal ventilator, the chest was opened along the midline of the mouse chest, and on the epicardial surface of the left ventricle, a 6 × 6 electrode microelectrode (electrode impedance: 1.5–1.7Q, PA03606060101, multielectrode probe array) was used to record epicardial activation electrical mapping. Data were recorded using multichannel systems (EMS64-USB-1003, MappingLab Ltd., Oxford, UK). Date of the conduction velocity (CV), the inhomogeneity index, and the absolute inhomogeneity were calculated using EMapScope 4.0 software (MappingLab Ltd., Oxford, UK).

### 2.11. Blood Pressure

The mice were placed in animal holders on a far-infra-red warming platform, and their body temperatures were maintained at 37 °C. The blood pressure of conscious mice was measured using volume pressure-recording sensor equipment (BP-98-AL, Softron, Tokyo, Japan). After a period of stabilization, systolic blood pressure (SBP), diastolic blood pressure (DBP), and mean blood pressure (MBP) were meticulously monitored.

### 2.12. Transmission Electron Microscope

The mouse left ventricular tissue samples were fixed in 2.5% glutaraldehyde (pH = 7.4) for 2 h. After being washed three times with 0.1 M phosphate buffer (pH = 7.2) and fixed in 1% osmic acid at 4 °C for 2 h, the samples were then gradient dehydrated in a graded series of ethanol. Subsequently, the samples were embedded in Epon-Araldite resin for penetration and placed in a model for polymerization. After the semi-thin section was used for positioning, the ultrathin section was made and collected for microstructure analysis, followed by the counterstaining of 3% uranyl acetate and 2.7% lead citrate. They were then observed with a HT7800 (HITACHI, Tokyo, Japan) transmission electron microscope.

### 2.13. Histology

After the ventricular tissue was wax-sealed and embedded in an automatic dehydrator, the tissue information was marked, and the 5 μm thick slice was maintained for serial sectioning. Selected intact and fold-free tissue sections were collected and laid flat in a spreader. After each tissue section was fully unfolded, it was quickly picked up with a glass slide, put in a 65 °C oven for 1 h, and then in the section box for preservation. The tissues were later stained to observe the morphology.

### 2.14. Cell Culture and Cell Experiments

The H9c2 cell line was used for the in vitro studies and maintained in a DMEM high-glucose culture medium (supplemented with 100 U/mL/100 mg/mL pen/strep) containing 10% FBS at 37 °C, and in a 5% CO_2_ cell culture incubator with medium changes every other day. Cells were divided into four groups: control (PBS), DOX (1 μM for 24 h), DOX + Fer-1 (1 μM), and DOX + CeO_2_@BSA NPs (200 μM measured by cerium).

### 2.15. Cellular Mitochondrial Membrane Potential and ROS Measurement of H9c2 Cell Line

The H9c2 cells were incubated with JC-1 staining buffer in the dark for 20 min at 37 °C and washed twice with PBS. The processes of measurement were used with an Olympus FV1000 LSCM under the excitation wavelengths of 488 nm and 543 nm. The decreased relative fluorescence proportion of aggregate JC-1 (red)/monomeric JC-1 (green) indicates a decrease in the mitochondrial membrane potential (MMP). ROS were detected using a DCFH-DA fluorescent probe. The cells were incubated with 10 μM DCFH-DA in PBS in the dark for 20 min at 37 °C. Fluorescence was determined at 488 nm for excitation and 525 nm for emission. Microscope images were saved as TIFF files and processed for densitometric quantification with ImageJ version 1.46 (NIH). The software settings were kept the same for every image analyzed.

### 2.16. Mitochondrial Ferrous Ions and Mitochondrial Lipid Peroxidation Toxicity Assay

The H9c2 cells were incubated with Mito-FerroGreen (M489 Dojindo Laboratories, Kumamoto Prefecture, Japan) or MitoPeDPP (M466, Dojindo Laboratories, Kumamoto Prefecture, Japan) at 37 °C for 30 min out of the light. After incubation, the cells were washed with Hanks’ HEPES buffer, and the fluorescence was measured with an Olympus FV1000 LSCM under excitation and emission wavelengths of 470 nm and 525 nm.

### 2.17. Western Blotting

The cardiac tissues were mixed in lysis buffer containing protease and phosphatase inhibitors. The supernatant was collected via centrifugation at 12,000 rpm and 4 °C for 20 min. The concentrations of proteins were measured using the BCA assay kit (Thermo Scientific, Waltham, MA, USA). After protein samples were immobilized, 30 ug of total protein per sample was separated on SDS-PAGE, electrophoresed, and transferred to PVDF membranes. The membranes were blocked in 5% nonfat milk (20 mM Tris HCl, pH = 7.6, 137 mM NaCl, 0.05% Tween 20) for 1 h at room temperature, and then incubated at 4 °C overnight with the following primary antibodies: α-Tubulin (1:1000, K006154P, Solarbio), GPX4 (1:1000, ab125066, Abcam, Cambridge, UK), mitochondrial transcription factor A (mtTFA, 1:1000, ab252432, Abcam), the α subunit of peroxisome proliferator-activated receptor-γcoactivator-1 (PGC-1α, 2 µg/mL, ab106814, Abcam), and dynamin-related protein 1 (DRP1, 1:1000, ab184247, Abcam). Next, the membranes were incubated with horseradish peroxidase-conjugated secondary antibody (1:1000, Proteintech, Rosemont, IL, USA) at room temperature for 1 h, and then the membranes were treated with chemiluminescent detection reagent and exposed to film using a Tanon 5200 Multi Chemiluminescent Imaging System (Tanon Science & Technology Co., Ltd., Shanghai, China). Densitometric analysis was completed using Image J software (Fiji—ImageJ, NIH Image, Bethesda, MD, USA).

### 2.18. Biodistribution and Biocompatibility Assessment

The healthy C57BL/6J male mice were intravenously administered with CeO_2_@BSA normal saline solution (intravenous administration of 1 mg/kg measured by cerium for a total of four doses, n = 3) and the mice were sacrificed at the end of experiment. The major organs were collected, and then stained with HE to observe the morphological changes, and the remaining tissues were weighed and dissolved in a concentrated nitric acid solution. The amount of Ce elements in the different samples was analyzed by ICP-MS. In addition, blood samples were collected for hematological and biochemical analysis before the animals were euthanized.

### 2.19. Statistical Analysis

The data are presented as mean ± standard error of mean (SEM). The normal distribution was checked with the Kolmogorov–Smirnov test. Data corresponding to normal distributions were analyzed statistically using one-way analysis of variance (ANOVA), followed by the LSD test. Non-parametric tests were used for non-normally distributed data. The differences between the two groups were analyzed using the t test. All statistical analyses were performed using SPSS (version 26.0, International Business Machines Corporation, Armonk, NY, USA) or GraphPad Prism (version 8.0, GraphPad, San Diego, CA, USA) software for statistical charts and group charts, and *p* of < 0.05 was considered statistically significant.

## 3. Results and Discussion

### 3.1. CeO_2_@BSA NP Characterization

**Figure 2:**
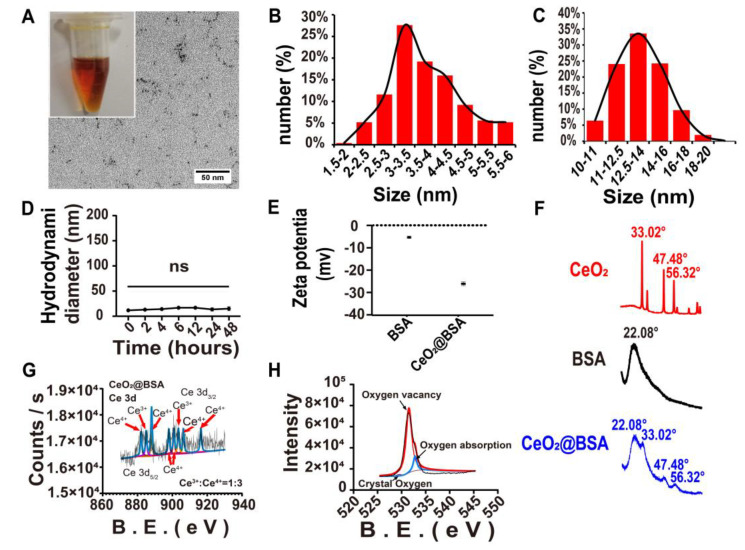
CeO_2_@BSA NP characterization. (**A**) Appearance and TEM images (Scale bar = 50 nm) of NPs. (**B**) Size distribution of nanoparticles as shown in (**A**). (**C**) Diameter distribution of nanoparticles measured via DLS. (**D**) Particle size at different points in 48 h measured by DLS. (**E**) Zeta potential of BSA and NPs. (**F**) XRD of CeO_2_, BSA, and NPs. (**G**) XPS of the spectrum of the Ce3d. (**H**) XPS of the spectrum of the O1s. TEM: transmission electron microscopy, DLS: dynamic light scattering, XRD: X−ray diffraction, XPS: X−ray photoelectron spectroscopy. Data are expressed as mean ± SEM of three independent replicates.

The CeO_2_@BSA NP solution had a clear and transparent brownish-yellow appearance after BSA biomineralization, though CeO_2_ was insoluble in water (Figure 2A). Transmission electron microscopy (TEM) images revealed the encapsulated CeO_2_@BSA was ≈ 3 nm in diameter (Figure 2A,B). The CeO_2_@BSA NPs showed an average hydrodynamic diameter of ≈14 nm from dynamic light scattering (DLS, Figure 2C). The stability of the CeO_2_@BSA NPs was evaluated by monitoring the NPs’ size change using DLS at different time points at 4 °C (Figure 2D), in different solvents (Figure S1A), and at different pH levels (Figure S1B). Next, the zeta potential of the BSA remained stable (from −6.627 mV to −32.40 mV, Figure 2E) after the reaction. Moreover, for phase analysis, the XRD pattern analysis (Figure 2F) exhibited three prominent peaks characteristic of cerium oxide, corresponding to the (111)-, (220)-, and (311)-planes (JCPDS: No.01-078-3080), respectively, in the cubic fluorite crystal structure. This suggests the existence of CeO_2_ within the BSA protein templates. XPS revealed the hybrid nature of Ce (III) to Ce (IV) ions, with corresponding binding energy peaks for Ce^3+^ (885.00 ev and 903.50 ev) and Ce^4+^ (882.10 ev, 888.10 ev, 898.00 ev, 900.90 ev, 906.40 ev, and 916.35 ev) in the spectrum of the Ce3d (Figure 2G), and the ratio between them was roughly 1:3, which almost coincided with the spectrum of a previous study [51]. Meanwhile, the spectrum of the O1s (Figure 2H) showed many oxygen vacancies in CeO_2_@BSA NPs, which enhanced their effectiveness in catalyzing ROS.

### 3.2. In Vitro Hybrid Enzymatic Mimetic Activity of CeO_2_@BSA NPs

**Figure 3:**
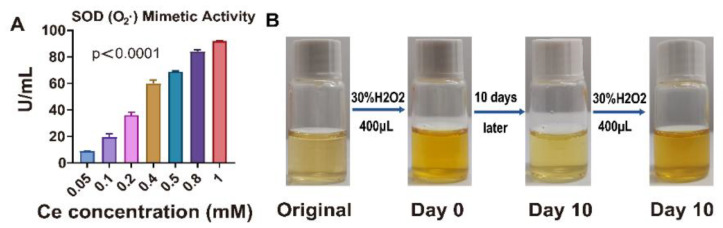
Superoxide dismutase (SOD)- and catalase (CAT)-hybrid-mimetic enzyme activities of CeO_2_@BSA NPs in vitro. (**A**) Statistical graphs of SOD-mimetic enzyme activity of CeO_2_@BSA NPs. (**B**) The digital images showed the coloration of CeO_2_@BSA NP solutions moments after the addition of H_2_O_2_. Ce^3+^ redox state is peculiar to colorless ions and the coloration is peculiar to the Ce^4+^ redox state. A change in color indicates a change in Ce^4+^/Ce^3+^. H_2_O_2_: hydrogen peroxide. The data are expressed as mean ± SEM of three independent replicates.

We next found that CeO_2_@BSA NPs had excellent SOD-mimetic (Figure 3A) and CAT-hybrid-mimetic enzyme (Figure 3B and Figure S2) activities, which were dose-dependent in vitro. The Ce^3+^ redox state is peculiar to colorless ions and the coloration is peculiar to the Ce^4+^ redox state. A color change in the NP solution after the addition of hydrogen peroxide indicates a change in the Ce^4+^/Ce^3+^ state, showing that a redox reaction took place [28]. After ten days, the solution turned colorless and another color change took place after the addition of hydrogen peroxide, demonstrating the existence of Ce^4+^/Ce^3+^ redox cycling.

### 3.3. CeO_2_@BSA NPs Protected DIC by Preventing Ferroptosis in Cardiomyocytes

**Figure 4:**
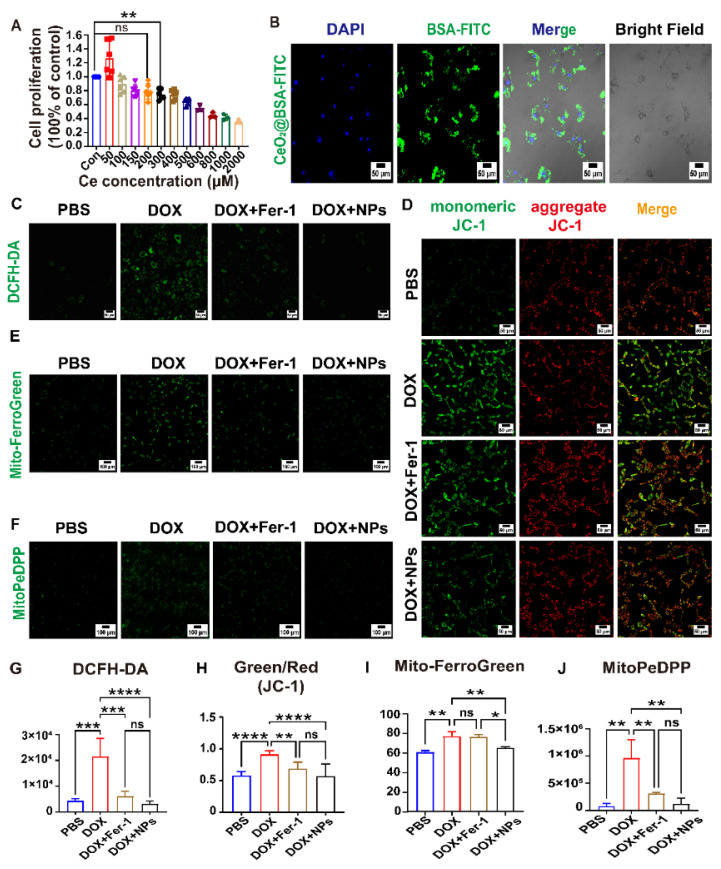
CeO_2_@BSA NPs protect against DIC by preventing ferroptosis in H9c2 cell lines. (**A**) MTT cell viability experiment of the different concentrations of CeO_2_@BSA NPs measured by cerium concentration on H9c2 cells at 24 h. (**B**) Representative confocal microscopy images of the cellular uptake of CeO_2_@BSA NPs at 6hrs, scale bar = 100 μm. (**C**) Fluorescent images of H9c2 cells that were stained with DCFH-DA, scale bar = 50 μm. (**D**) Representative confocal microscopy images of H9c2 cells that were stained with JC-1 dye, scale bar = 50 μm. (**E**) Representative fluorescent images of H9c2 cells’ mitochondrial ferrous ions that were stained with Mito-FerroGreen, scale bar = 100 μm. (**F**) Representative fluorescent images of H9c2 cells’ mitochondrial lipid peroxides that were stained with MitoPeDPP, scale bar = 100 μm. (**G**) Quantification of ROS via DCFH-DA intensity in (**C**). (**H**) Quantification of MMP of H9c2 cells via JC-1 monomer/JC-1 aggregates in (**D**). (**I**) Quantification of ferrous ions in mitochondria measured by Mito-FerroGreen intensity in (**E**). (**J**) Quantification of mitochondrial lipid peroxides by MitoPeDPP intensity in (**F**). DAPI: 4′,6′-diamidino-2-phenylindole, FITC: fluorescein isothiocyanate, DOX: doxorubicin, Fer-1: ferrostatin-1, NPs: CeO_2_@BSA nanoparticles, PBS: phosphate buffer solution, H_2_O_2_: hydrogen peroxide; DCFH-DA: 2′,7′-dichlorofluorescin diacetate; ROS: reactive oxygen species; MMP: mitochondrial membrane potential. Data are expressed as mean ± SEM of three to five independent replicates; * *p* < 0.05, ** *p* < 0.01, *** *p* < 0.001, **** *p* < 0.001, ns: no statistical difference.

To further evaluate the potential for biomedical applications, the methyl thiazolyl tetrazolium (MTT) assay showed that the viability of H9c2 cells were not influenced by CeO_2_@BSA NPs, even at high doses of Ce of up to 200 μM (Figure 4A). This suggested the great biocompatibility of the NPs. We confirmed that the CeO_2_@BSA NPs were taken up by the H9c2 cells in large quantities (Figure 4B). Additionally, the MTT assay showed that 1 μM DOX significantly inhibited cellular activity after a 24 h co-incubation (Figure S3).

The loss of cardiomyocytes is the major cause of DOX-induced cardiac injury and dysfunction. Therefore, the prevention of cardiomyocyte loss was an effective approach to the treatment of DIC [52]. DCFH-DA fluorescence found that the intracellular ROS level was significantly higher in the DOX group compared with the PBS group and the ROS level was markedly decreased both in the CeO_2_@BSA and Fer-1 group (Figure 4C,G). Mitochondrial membrane potential (MMP, ΔΨm) is commonly used to measure mitochondrial function, and a loss of ΔΨm indicated mitochondrial dysfunction [53]. MMP was quantified using the JC-1 dye. The MMP was significantly reduced in the DOX group and improved both in the CeO_2_@BSA and Fer-1 group (Figure 4D,H).

Ferroptosis was distinctly different from other known cell death modalities in terms of cell morphology, genetics, and biochemistry. The main feature of ferroptosis was an iron-dependent lethal accumulation of lipid peroxidation and ROS accumulation. Free intracellular iron ions were in the form of stable Fe^2+^ and Fe^3+^. Mito-FerroGreen is a new fluorescent probe for the detection of the ferrous ion (Fe^2+^) in mitochondria, which is the site of iron–sulfur clusters and hemoglobin synthesis. Mito-FerroGreen fluorescence was significantly strong in the DOX group and markedly decreased in the CeO_2_@BSA group, but Fer-1 did not significantly reverse the production of mitochondrial ferrous ions (Figure 4E,I). MitoPeDPP is a novel fluorescent dye that could cross cell membranes and accumulate on the inner mitochondrial membrane due to its triphenylphosphine structure, which could be oxidized by lipid peroxides to emit strong fluorescence, indicating the occurrence of mitochondria-dependent ferroptosis [31]. MitoPeDPP fluorescence was significantly strong in the DOX group and markedly decreased both in the CeO_2_@BSA and Fer-1 groups (Figure 4F,J). The above results shows that the CeO_2_@BSA NPs could decrease ROS, suppress ferroptosis, and improve mitochondrial function at the cellular level. The results of the study also showed that the CeO_2_@BSA NPs outperformed Fer-1.

### 3.4. Preventive and Therapeutic Efficacy of CeO_2_@BSA NPs on Doxorubicin-Induced Cardiac Structural and Electrical Remodeling in Mice

**Figure 5:**
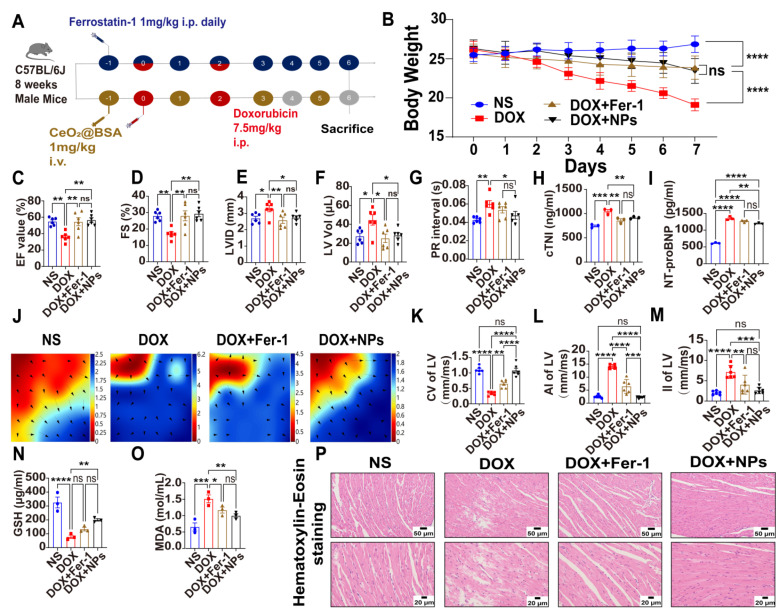
Therapeutic effects of CeO_2_@BSA NPs on doxorubicin-induced cardiac dysfunction in mice. (**A**) Schematic animal experiment and intervention protocol. (**B**) Statistical chart of weight change in four groups. (**C**) Analysis results of LVEF in M-mode of echocardiographic images. (**D**) Analysis results of LVFS in M-mode of echocardiographic images. (**E**) Analysis results of LVID in M-mode of echocardiographic images. (**F**) Analysis results of LV Vol in (**C**). (**G**) Analysis results of PR interval of electrocardiography. (**H**) Statistical chart of serum cTnI in four groups. (**I**) Statistical chart of serum NT-proBNP in four groups. (**J**) Representative epicardial electrical mapping recorded for LV. (**K**) Conduction velocity of LV in (**J**). (**L**) Absolute inhomogeneity of LV in (**J**). (**M**) Inhomogeneity index of LV in (**J**). (**N**) Statistical chart of serum GSH in four groups. (**O**) Statistical chart of serum MDA in four groups. (**P**) Representative HE staining of myocardial tissue in four groups, scale bar = 50 μm or 20 μm. NS: normal saline, DOX: doxorubicin, Fer-1: ferrostatin-1, NPs: CeO_2_@BSA nanoparticles, LV: left ventricle, LVEF: left ventricular ejection fraction, LVFS: left ventricular fractional shortening, LVID: left ventricular internal diameter, LV Vol: left ventricular volume, cTnI: cardiac troponin I, NT-proBNP: N-terminal brain natriuretic peptide, GSH: glutathione, MDA: malondialdehyde, HE staining: Hematoxylin-eosin staining. Data are expressed as mean ± SEM of three to six independent replicates; * *p* < 0.05, ** *p* < 0.01, *** *p* < 0.001, **** *p* < 0.001, ns: no statistical difference.

The cardiotoxicity of DOX was mainly related to its cumulative dose of the drug and we performed mice modelling using a cumulative dose of 15 mg/kg, as recommended by the European Society of Clinical Oncology (ESMO) guidelines. The preventive and therapeutic interventions of CeO_2_@BSA NPs were administered through the tail vein and Fer-1 was administered intraperitoneally. The schematic animal experiment protocol is shown in Figure 5A. There was a significant decrease in body weight and a poor general condition of the mice in the DOX group, which was improved by the administration of Fer-1 and CeO_2_@BSA NPs (Figure 5B). Hemodynamics through the non-invasive monitoring of mice tails showed that the HR, SBP, DBP, and MBP were not significantly different between the four groups (Figure S4).

Echocardiograms showed abnormality of diastolic and systolic functions (Figure S5) in the DOX group, manifested by a decrease in the left ventricular ejection fraction (LVEF, Figure 5C), a decrease in the left ventricular fractional shortening (LVFS, Figure 5D), a larger left ventricular internal diameter (LVID, Figure 5E), and an expanded left ventricular volume (LV Vol, Figure 5F), which could be reversed by Fer-1 and CeO_2_@BSA NPs.

For the ECG data, there was a significantly longer PR interval in the DOX group and recovery in CeO_2_@BSA NPs (Figure 5G), despite the P wave duration, QRS duration, and QT and RR interval being not significantly different across the four experimental groups (Figure S6).

Serum cTnI and NT-proBNP are recognized markers of cardiomyocyte and cardiac impairment [32]. They were significantly elevated in the DOX group, and decreased in the treatment group (Figure 5H,I).

In vivo epicardial electrical labelling (mapping images, Figure 5J) showed that the left ventricular conduction velocity (LVCV, Figure 5K) was significantly lower in the DOX group, accompanied by higher LV conduction dispersion (absolute, Figure 5L; index, Figure 5M), which was consistent with prior clinical trial findings [54] and may be the basis of malignant ventricular arrhythmias caused by DOX. However, the above abnormalities of cardiac electrical conduction were restored by Fer-1 and CeO_2_@BSA NPs, though CeO_2_@BSA NPs were significantly superior to Fer-1.

Glutathione is a tripeptide compound in the body that is involved in antioxidant and drug metabolism and is usually present in a reduced state (GSH) [33]. MDA is a compound formed after the breakdown of lipid peroxides [55]. Both are widely used as markers of oxidative stress and ferroptosis. In line with previous studies, GSH was significantly lower in the DOX group and recovered significantly in the CeO_2_@BSA NP group, but did not reach statistical significance in the Fer-1 group (Figure 5N). Meanwhile, MDA was significantly higher in the DOX group and reduced significantly in both treated groups (Figure 5O).

Hematoxylin/eosin (HE) staining was used to evaluate morphological changes in the myocardium. The results showed that compared with the NS group, the myocardial structure of the ventricular tissue in the DOX group was destroyed, and the cardiomyocytes were extensively vacuolated and arranged in a disorderly manner. All these abnormal changes were, however, reversed in the CeO_2_@BSA and Fer-1 groups (Figure 5P).

Combining the above results, CeO_2_@BSA NPs could be a promising treatment candidate for reversing the cardiac structural and electrical remodeling caused by DIC. A recent study has reported the use of non-drug interventions for the treatment of DIC. Prathumsap et al. [56] reported that vagus nerve stimulation (VNS) exerted cardioprotective effects against DIC via the activation of both the muscarinic receptor (mAChR) and nicotinic acetylcholine receptor (nAChR). However, large clinical trials are needed to further verify the safety and efficacy.

### 3.5. CeO_2_@BSA NPs Exhibited Protective Effect against Ferroptosis by Restoring GPX4 Expression and Improving Mitochondrial Function and Homeostasis

**Figure 6:**
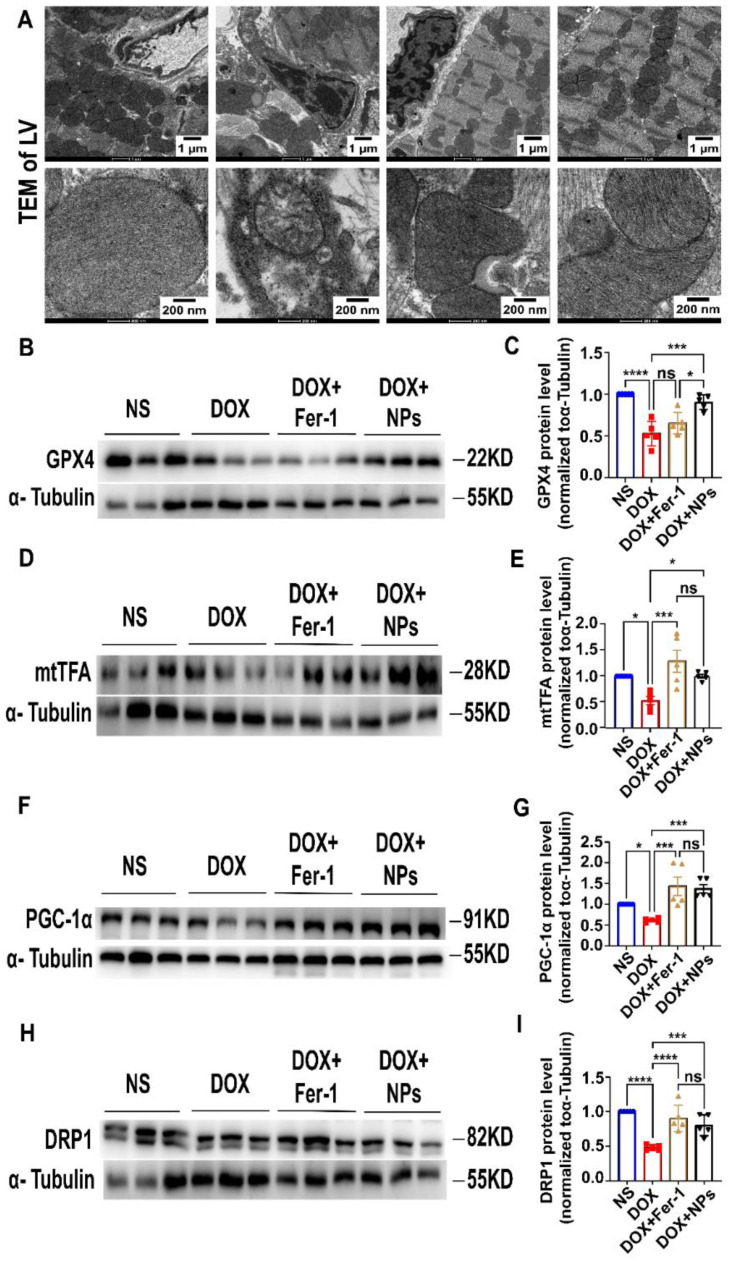
CeO_2_@BSA NPs exhibit a protective effect against ferroptosis by maintaining mitochondrial morphology, increasing GPX4, and mitochondria-related protein expression. (**A**) TEM images of mitochondria in mice cardiomyocytes, scale bar = 1 μm or 200 nm. (**B**) Representative Western blot results of GPX4 in the four groups. (**C**) Analysis of the protein expression of GPX4 in the four groups. (**D**) Representative Western blot results of mtTFA in the four groups. (**E**) Analysis of the protein expression of mtTFA in the four groups. (**F**) Representative Western blot results of PGC-1α in the four groups. (**G**) Analysis of the protein expression of PGC-1α in the four groups. (**H**) Representative Western blot results of DRP1 in the four groups. (**I**) Analysis of the protein expression of DRP1 in the four groups. NS: normal saline, DOX: doxorubicin, Fer-1: ferro-statin-1, NPs: CeO_2_@BSA nanoparticles, GPX4: glutathione peroxidase 4, mtTFA: mitochondrial transcription factor A, PGC-1α: α subunit of peroxisome proliferators-activated receptor-γcoactivator-1, DRP1: dynamin-related protein 1. Data are expressed as mean ± SEM of three to five independent replicates; * *p* < 0.05, *** *p* < 0.001, **** *p* < 0.001, ns: no statistical difference.

Mitochondria are important organelles in the DOX-induced ferroptosis of cardiomyocytes [26,57]. Oh et al. reported that mitochondrial events could be the ultimate step in determining the final cell fate [58]. Smaller mitochondria, increased bilayer density, and the reduced cristae of mitochondria in cardiomyocytes were observed by TEM in the DOX group, suggesting mitochondrial-dependent ferroptosis that was consistent with previous studies [25,59]. Additionally, the mitochondrial injury was, however, reversed by Fer-1 and CeO_2_@BSA NPs (Figure 6A).

The GPX4 attenuated lipid peroxide toxicity and maintained membrane lipid bilayer homeostasis to inhibit ferroptosis due to its catalytic activity. The inactivation of GPX4, however, led to a disrupted oxidative balance and disrupted membrane structure, provoking ferroptosis. Because of its specific mechanism of action, GPX4 was considered a ferroptosis core regulator [60]. Consistent with this, we evaluated the expression of GPX4 and the mitochondria-associated protein of the myocardial tissues and found that the expression levels of GPX4, mtTFA, PGC-1α, and DRP1 were significantly lower in the DOX group compared with the NS group (Figure 6B–I). The above abnormalities were, however, significantly restored in the CeO_2_@BSA and Fer-1 groups, except for GPX4 protein expression which was not statistically different compared to the Fer-1 group.

Mitochondrial homeostasis is important for maintaining the normal functions of mitochondria [61,62]. The mtTFA protein is bound to the mitochondrial light strand promoter to participate in mitochondrial transcription regulation, which is required for the maintenance of normal levels of mitochondrial DNA [63]. The PGC-1α is a master controller that promotes the transcription of the genes involved in mitochondrial biogenesis and oxidative metabolism, hence enhancing fatty acid β-oxidation, oxidative phosphorylation, and ATP production [64]. The DRP1 is required for mitochondrial fission during mitosis and plays a role in mitochondrial and peroxisomal division [65].

Recently, Wang et al. [66] reported that DOX can significantly affect the pathways related to the energy metabolism of myocardial cells via metabonomic analysis. Additionally, Di’ao Xinxuekang (DXXK) could improve DIC in mice by inhibiting ferroptosis through the AMPK-mediated energy protection pathway. He et al. [67] reported that epigallocate-chin-3-gallate pretreatment could effectively decrease iron accumulation, inhibit oxidative stress and abnormal lipid metabolism, and thereby alleviate DIC ferroptosis by upregulating AMPKα2 and activating adaptive autophagy. These results suggest that our future research should focus more on mitochondrial energy metabolism.

To summarize, the above results reveal that the cardioprotective effects of CeO_2_@BSA NPs against DIC may be mediated by the restoring of GPX4 expression, thereby maintaining mitochondrial homeostasis and function, as well as restoring mitochondria-dependent ferroptosis. The results of the study also provide some insights into the role of ferroptosis in DIC.

### 3.6. In Vivo Metabolism and Biocompatibility of CeO_2_@BSA NPs

**Figure 7:**
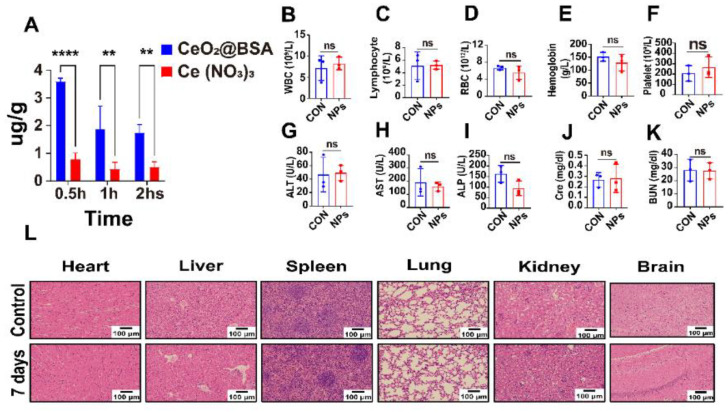
In vivo metabolism and biocompatibility of CeO_2_@BSA NPs. (**A**) Cardiac uptake of CeO_2_@BSA NPs and the equivalent cerium content of Ce(NO_3_)_3_ at different time points after tail vein injection. (**B**–**F**) Analysis of the blood routine examination of WBC (**B**), lymphocytes (**C**), RBC (**D**), hemoglobin (**E**), and platelets (**F**) in the two groups. (**G**–**I**). Analysis of the liver function via ALT (**G**), AST (H), and ALP (**I**) in the two groups. (**J**,**K**) Analysis of the renal function via Cre (**J**) and BUN (**K**) in the two groups. (**L**). Representative HE staining of heart, liver, spleen, lung, kidney, and brain in the two groups after a week of treatment, scale bar = 100 μm. WBC: white blood cell, RBC: red blood cell, ALT: alanine transaminase, AST: aspartate transaminase, ALP: alkaline phosphatase, Cre: creatinine, BUN: blood urea nitrogen, HE staining: hematoxylin/eosin (HE). Data are expressed as mean ± SEM of three independent replicates; ** *p* < 0.01, **** *p* < 0.001, ns: no statistical difference.

The biocompatibility of CeO_2_@BSA NPs was also evaluated systematically following its administration via the tail vein. The cerium content in the major organs was determined via ICP-MS analysis. The metabolism of CeO_2_@BSA NPs in the heart showed a persistent presence compared to the equivalent cerium content of Ce(NO_3_)_3_ (Figure 7A and Figure S7), suggesting a high stability provided by biomimetic mineralization. Meanwhile, the metabolism of cerium in the major tissues and organs showed that it was mainly metabolized by the hepatobiliary systems (Figure S8) in line with a prior study [68]. The routine blood examination (Figure 7B–F), liver function test (Figure 7G–I), renal function test (Figure 7J–K), morphological changes in the major organs (Figure 7L), and absence of obvious body-weight loss (Figure S9) after a week post-injection showed that CeO_2_@BSA has excellent biocompatibility and low toxicity. This could be exploited to develop a novel cardiomyocyte ferroptosis protector to mitigate DIC in cancer patients and improve their prognosis and quality of life.

### 3.7. Study Limitations

There are some limitations associated with the present study. Firstly, we did not block the GPX4 pathway of mitochondrial oxidation in vivo or in vitro; thus, we could not further clarify that it was the sole signaling pathway to improve mitochondrial function by CeO_2_@BSA NPs. Secondly, we only assessed the effects of CeO_2_@BSA NPs on cardiomyocytes, whereas the potential effects of NPs on fibroblasts, immunocytes, mesothelium, endotheliocytes, or intercellular communication were not investigated [69,70,71,72].

## 4. Conclusions

The current study showed that the biomimetic mineralization of hybrid CeO_2_-based nanozymes against DIC were mediated by the effect of antioxidant stress and the restoration of GPX4 expression, thereby maintaining mitochondrial homeostasis and function, and restoring mitochondria-dependent ferroptosis. This provides some insights into the role of ferroptosis in DIC. Furthermore, the nanozyme could be a promising prevention and treatment candidate for clinical translation as a novel cardiomyocyte ferroptosis protector to mitigate DIC and improve the prognosis and quality of life of cancer patients.

## Data Availability

Not applicable.

## Supplementary Materials

The following supporting information can be downloaded at: https://www.mdpi.com/article/10.3390/nu15051090/s1, Figure S1: Particle size of CeO_2_@BSA nanoparticles in different solvents and different pH levels measured by dynamic light scattering; Figure S2: Digital images of catalase mimetic enzyme of CeO_2_@BSA nanoparticles; Figure S3: The effect of DOX on cellular viability; Figure S4: Analysis of the results of hemodynamics through non-invasive monitoring of mice tail; Figure S5: Representative electrocardiography recorded for the mice; Figure S6: Representative electrocardiography recorded for the mice in the four groups and analysis results of the P wave duration, QRS duration, QT interval, and RR interval. Figure S7: Metabolism of CeO_2_@BSA nanoparticles in the heart at different times of the day; Figure S8: Metabolism of CeO_2_@BSA nanoparticles in the major tissues and organs at different times of the week; Figure S9: Body-weight change in CeO_2_@BSA nanoparticles and normal saline groups.
