# Supplementary material for: Biomimetic Nanozymes Suppressed Ferroptosis to Ameliorate Doxorubicin-Induced Cardiotoxicity via Synergetic Effect of Antioxidant Stress and GPX4 Restoration
Source: Nutrients. 2023 Feb 22;15(5):1090. doi: 10.3390/nu15051090 (PMC10005374; doi:10.3390/nu15051090)
Supplement: Supplementary file 1 [file nutrients-15-01090-s001.zip › nutrients-2224811-supplementary.pdf]

# Supporting Information

# Biomimetic Nanozymes Suppressed Ferroptosis to Ameliorate Doxorubicin-induced Cardiotoxicity via Synergetic Effect of Antioxidant Stress and GPX4 Restoration

**Yunpeng Zhang [1, 2, †], Shuang Liu [3, †], Jing Peng [3, †], Shifeng Cheng [3], Qingling Zhang [1], Nan Zhang [1], Zandong Zhou [1], Yue Zhang [1,2], Yang Zhao [3, *], Tong Liu [1,2, *]**

1. Department of Cardiology, The Second Hospital of Tianjin Medical University, Tianjin 300211, People's Republic of China
2. Tianjin Key Laboratory of Ionic-Molecular Function of Cardiovascular disease, Tianjin Institute of Car-diology, The Second Hospital of Tianjin Medical University, Tianjin 300211, People's Republic of China
3. Department of Radiology, The Second Hospital of Tianjin Medical University, Tianjin 300211, People's Republic of China

*** Correspondence:** Tong Liu, M.D., Ph.D., E-mail: liutong@tmu.edu.cn. Tel.: +86-022-88328617. The Second Hospital of Tianjin Medical University, No.23, Pingjiang Road, Hexi District, Tianjin 300211, People's Republic of China. Joint corresponding author: Yang Zhao, Ph.D., E-mail: yang.zhao@tmu.edu.cn

**† These authors contributed equally to this work.**

# Table of Contents


| | Page |
|---|---|
| Supporting Figures | S3-S11 |

## Supporting figures

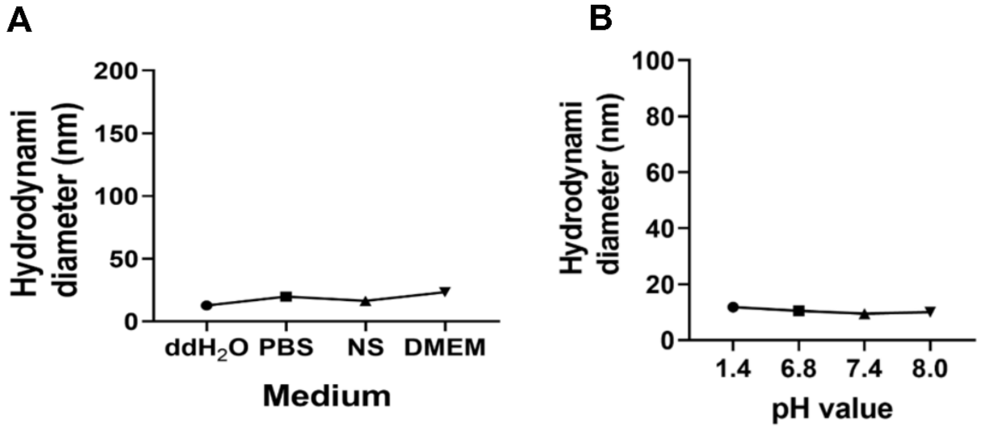


Figure S1: (A) Particle size in different solvents measured by dynamic light scattering (DLS) at 25ºC. (B) Particle size at different pH levels measured by DLS at 25ºC.

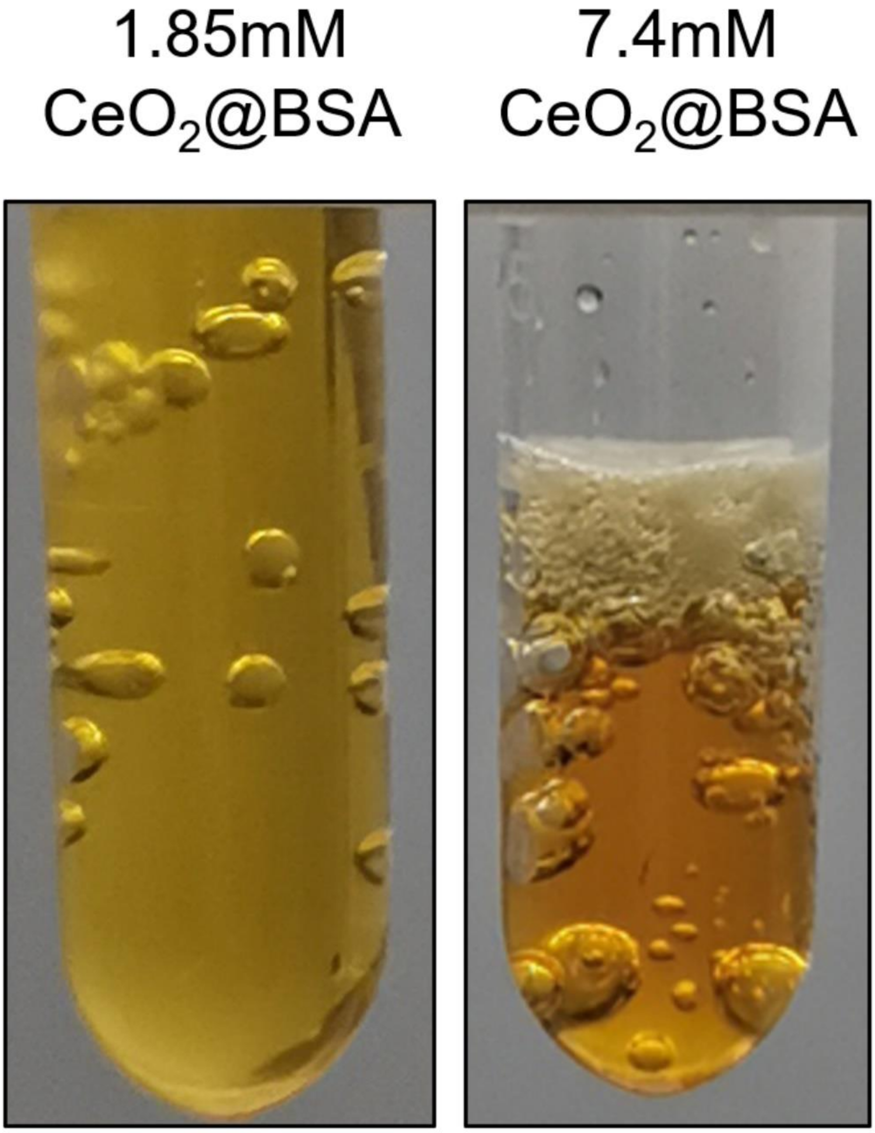


Figure S2: Digital images showed $CeO_2$@BSA nanoparticles catalyzed the production of oxygen from hydrogen peroxide and the degree of response was dose-dependent.

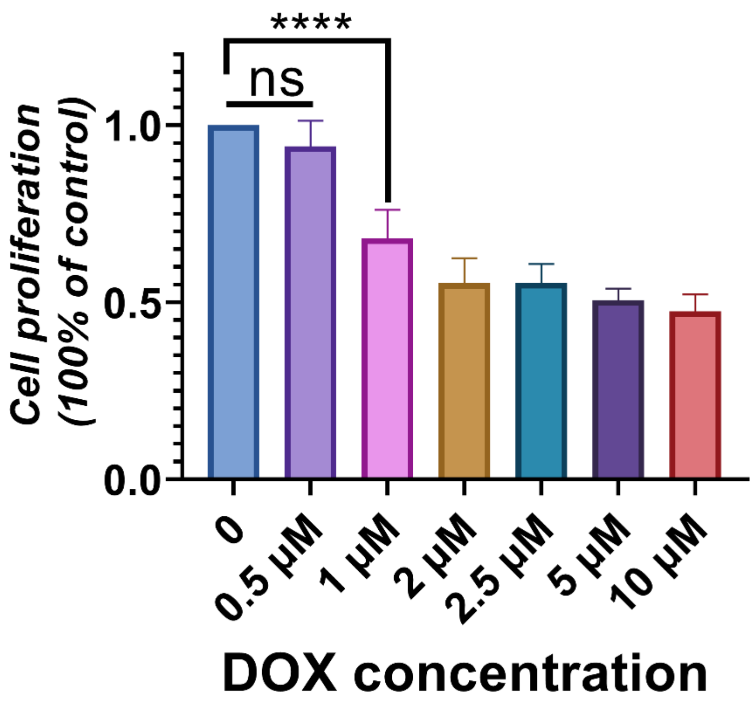


Figure S3: MTT cell viability experiment of different concentrations of doxorubicin (DOX) on H9c2 cells at 24hrs. Data are expressed as mean ± SEM of five independent replicates; ****P< 0.001, ns: no statistical difference.

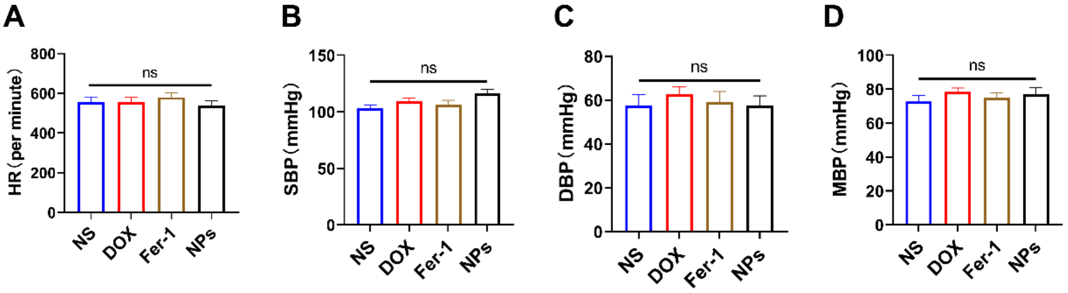


Figure S4: Analysis of the results of hemodynamics through non-invasive monitoring of mice tail in the four groups on the heart rate (HR, A), systolic blood pressure (SBP, B), diastolic blood pressure (DBP, C), and mean blood pressure (MBP, D). NS: normal saline, DOX: doxorubicin, Fer-1: ferrostatin-1, NPs: $CeO_2$@BSA nanoparticles. Data are expressed as mean ± SEM of six independent replicates. ns: no statistical difference.

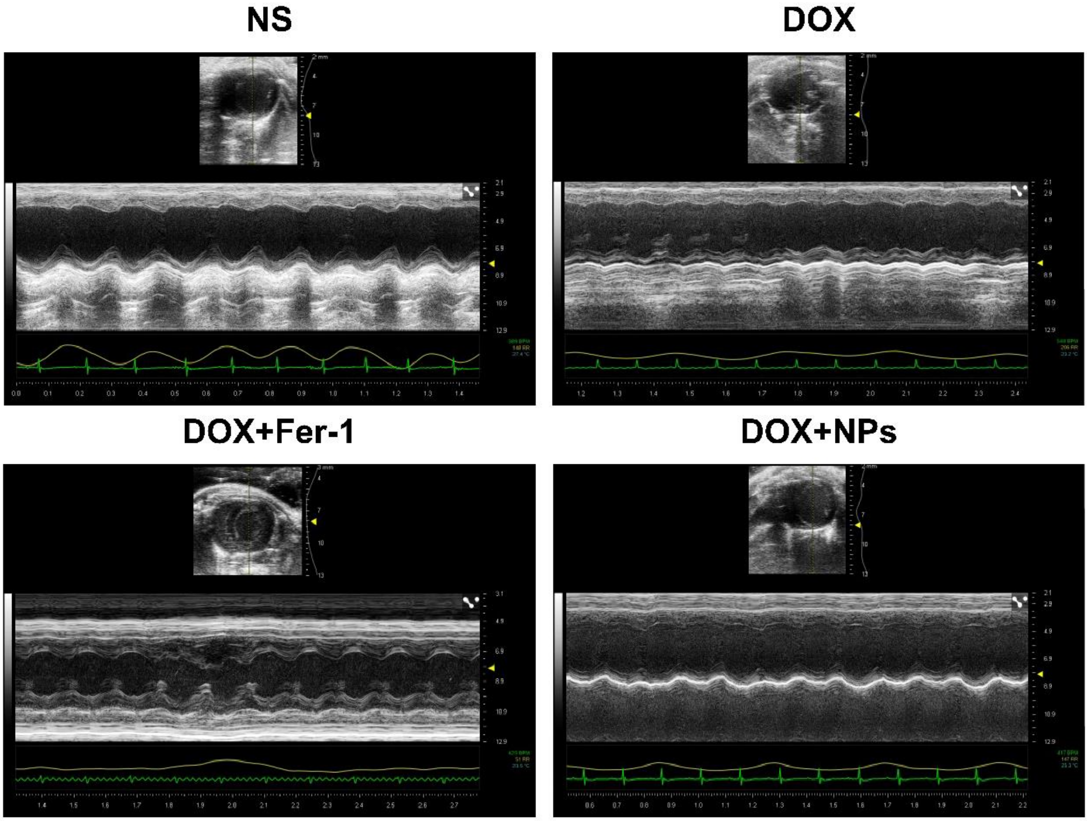


Figure S5: Representative two-dimensional and M-mode of echocardiographic images in four groups. NS: normal saline, DOX: doxorubicin, Fer-1: ferrostatin-1, NPs: $CeO_2$@BSA nanoparticles.

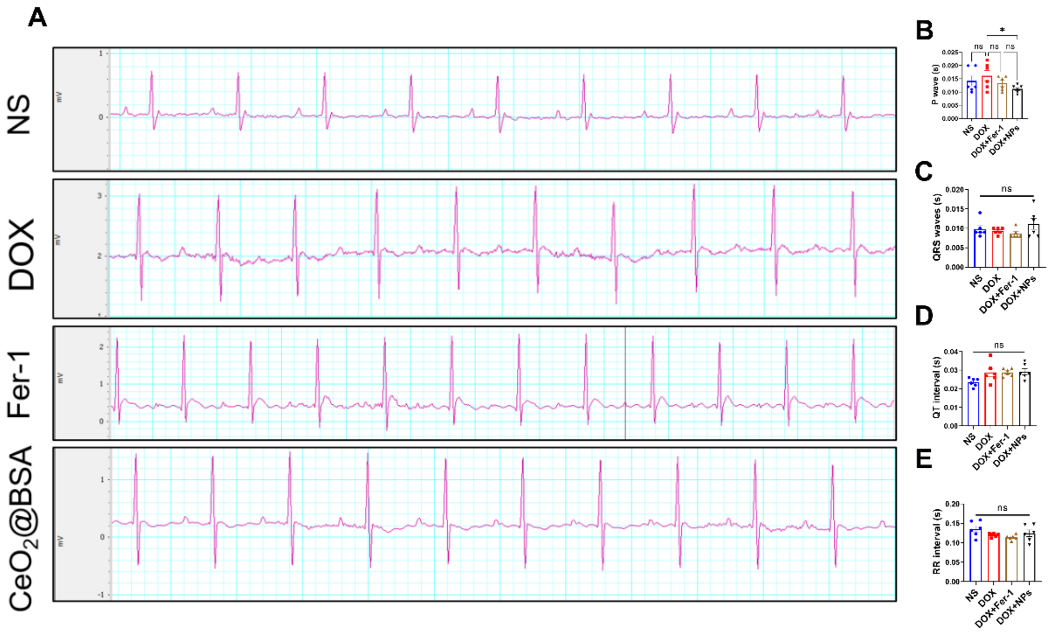


Figure S6: (A) Representative electrocardiography recorded for the mice in the four groups. (B-E) Analysis results of the P wave duration (B), QRS duration(C), QT interval (D), and RR(E) interval in (A). NS: normal saline, DOX: doxorubicin, Fer-1: ferrostatin-1, NPs: $CeO_2$@BSA nanoparticles. Data are expressed as mean ± SEM of six independent replicates. *$P < 0.05$, ns: no statistical difference.

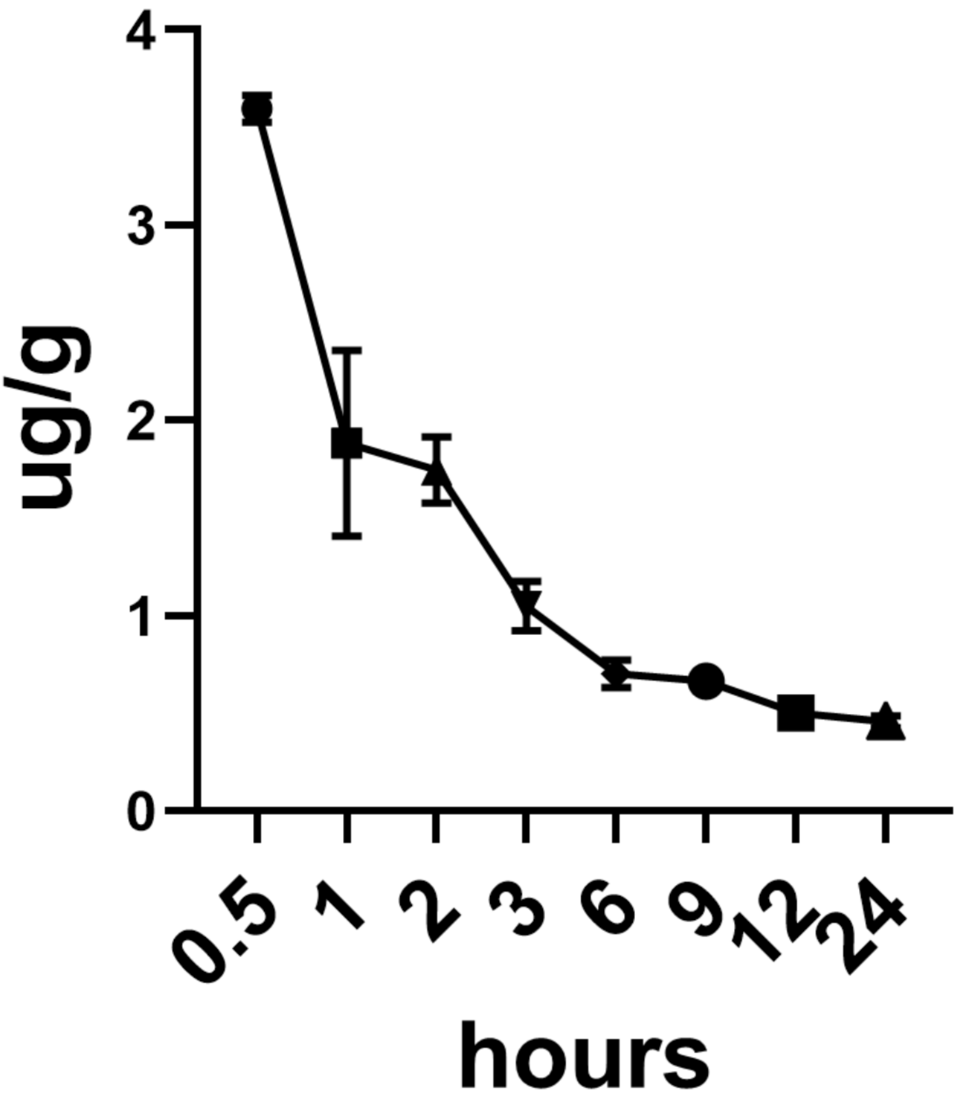


Figure S7: Metabolism of $CeO_2$@BSA nanoparticles in the heart at different times of the day. Data are expressed as mean ± SEM of three independent replicates.

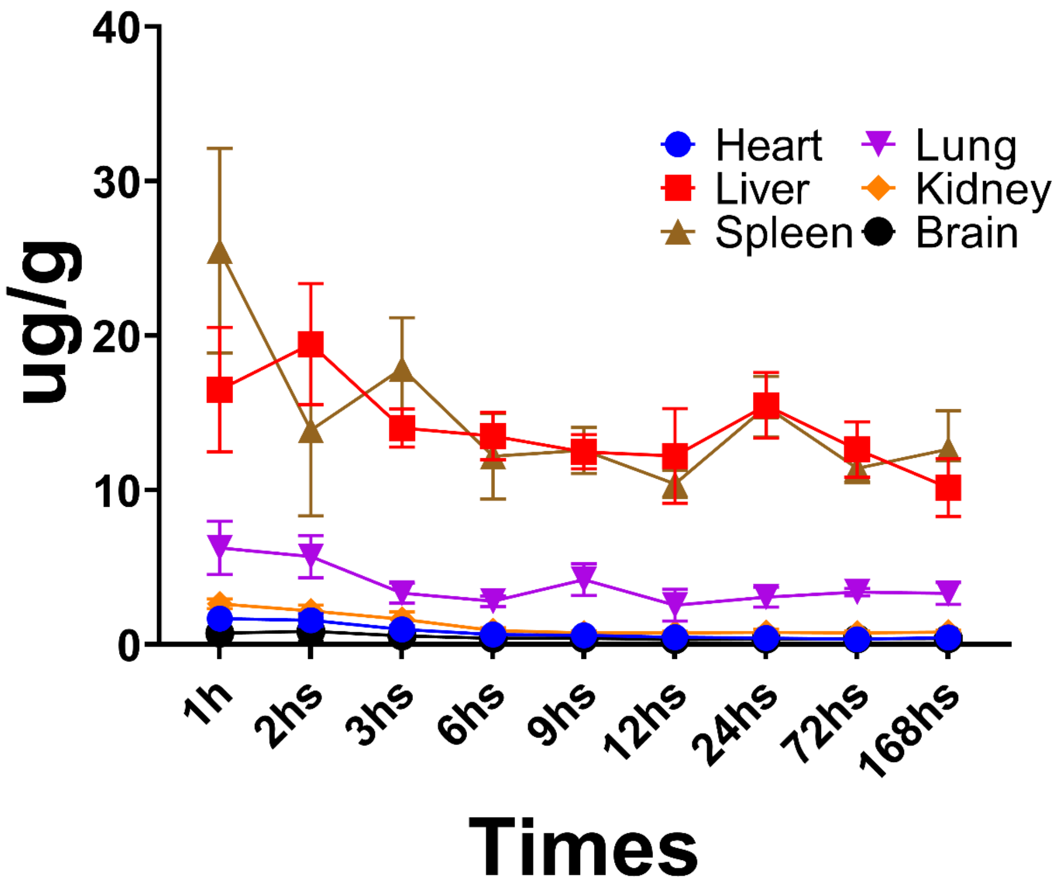


Figure S8: Metabolism of $CeO_2$@BSA nanoparticles in the major tissues and organs at different times of the week. Data are expressed as mean ± SEM of three independent replicates.

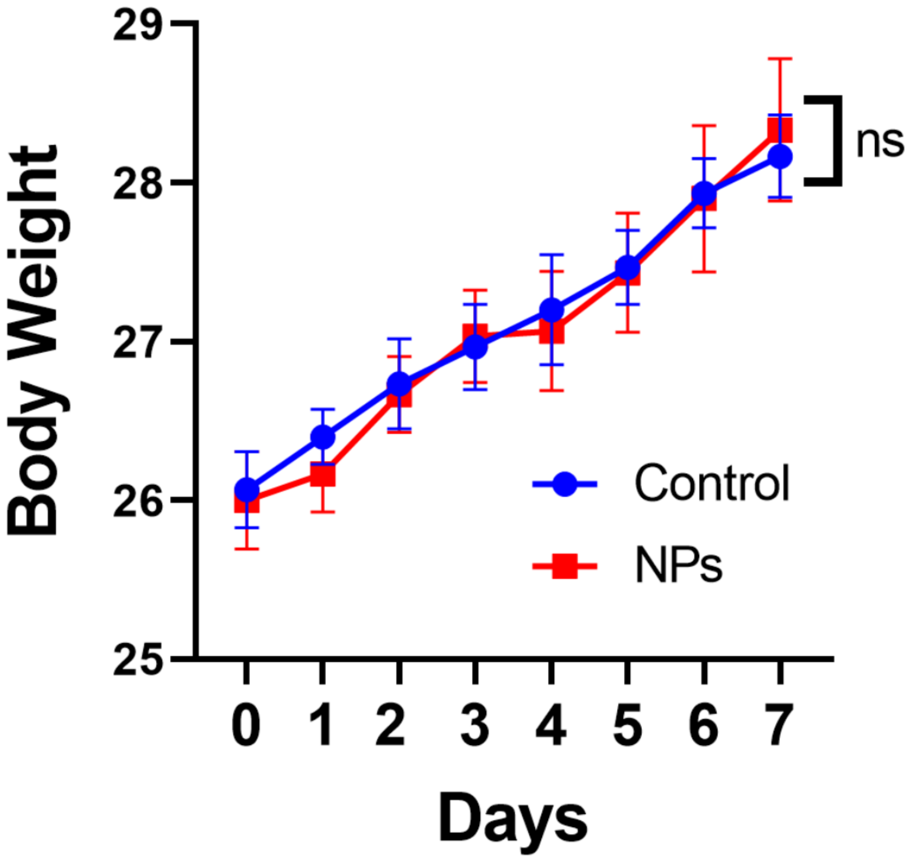


Figure S9: Body-weight change in two groups. NPs: $CeO_2$@BSA nanoparticles. Data are expressed as mean ± SEM of three independent replicates. ns: no statistical difference.
